# Oxygen Vacancy-Induced Strong Coordination of Carbon Dots with TiO_2_ for Enhanced Photocatalytic Hydrogen Production

**DOI:** 10.1021/acs.energyfuels.6c00577

**Published:** 2026-04-03

**Authors:** Mahdi Shahrezaei, Sergii Kalytchuk, Veronika Šedajová, Zdeněk Badura, Lukáš Zdražil, Morteza Afshar, Radek Zbořil, Stěpán Kment, Sourav Rej

**Affiliations:** † Regional Centre of Advanced Technologies and Materials, Czech Advanced Technology and Research Institute, 48207 Palacký University Olomouc, Šlechtitelů 241/27, 78371 Olomouc, Czech Republic; ‡ Nanotechnology Centre, Centre for Energy and Environmental Technologies, VSB−Technical University of Ostrava, 17. listopadu 2172/15, 708 00 Ostrava-Poruba, Czech Republic

## Abstract

Semiconductor nanomaterials decorated with low-cost and environmentally benign carbon dots (CDs) as cocatalysts have recently attracted increasing attention for a variety of photocatalytic reactions, driven by the main principles of circular economy and practical applications. Despite this progress, a mechanistic understanding of how semiconductor surface interacts with CDs to enhance the photogenerated charge separation still remains an open challenge. To unravel this, here, we meticulously designed two different hybrid systems: (i) CDs coupled with pristine brookite TiO_2_ nanorods (CDs/P–BTi) and (ii) CDs coupled with sonoreduced brookite TiO_2_ nanorods (CDs/R–BTi), synthesized via a simple hydrothermal procedure with optimized 0.5 wt % CD loading in each case. Interestingly, CDs/R–BTi shows a 10-fold higher photocatalytic H_2_ production rate as compared to sonoreduced brookite TiO_2_ nanorods (R–BTi). But CDs/P–BTi exhibits only 4-fold H_2_ production rate enhancement as compared to pristine brookite TiO_2_ nanorods (P–BTi). An in-depth mechanistic study confirms that the presence of oxygen vacancy-rich Ti^3+^ defect sites, together with the formation of strong interfacial heterojunctions, significantly enhances photogenerated charge carrier separation and their efficient migration to the respective catalytic centers in CDs/R–BTi, thereby resulting in the aforementioned higher rate enhancements. These results demonstrate that utilizing an oxygen vacancy-rich TiO_2_ surface is a more effective strategy than using a pristine TiO_2_ surface when CDs are used as cocatalysts for achieving high photocatalytic performance.

## Graphical abstract

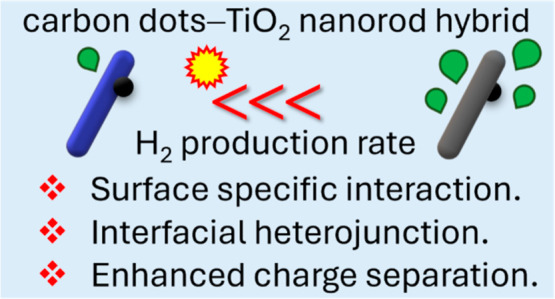

## 1 Introduction

The European Union is advancing toward climate-neutral energy systems to reduce greenhouse gas emissions, strengthen energy security, and decrease dependence on fossil fuel imports.^1–5^ As a part of this transition, the efficient production of hydrogen gas has gained strong attention as a clean energy carrier that can support decarbonization in areas such as renewable energy storage, transportation, and for chemical industry.^6^ To achieve these goals, it is essential to develop sustainable methods for hydrogen production using low-cost, scalable, renewable energy sources.^7–9^ Among the available technologies, solar-driven photocatalytic water splitting is considered as a promising direction for the production of hydrogen.^10–12^ However, practical implementation is still restricted by the low efficiency, weak absorption of sunlight, and material-related limitations of existing photocatalysts. Overcoming these issues is necessary for the realization of efficient and scalable photocatalytic hydrogen production in real-world applications.

Titanium dioxide (TiO_2_) coupled with noble metals such as Pt, Pd, and Rh acts as a high-performing, benchmark photocatalyst due to its favorable band-edge alignment for water splitting, efficient photogenerated separations with high solar-to-hydrogen conversion efficiency, and enhanced stability.^13–16^ But the limiting factor is the usage of noble metals, as their high cost, scarce availability, and geopolitical supply risks are incompatible with the long-term goals of sustainable and resilient hydrogen production to support the ever-growing demands of energy for the society. Recently, tremendous research development on single-atom catalysts paves the way for maintaining high efficiency with a very low amount of noble metals, thus reducing the catalyst cost significantly.^17–20^

In this context, carbon-based materials are undergoing rapid development and have attracted considerable attention as promising alternatives to noble-metal cocatalysts.^21–23^ Particularly, carbon dots (CDs) are carbon nanomaterials typically smaller than 10 nm and have emerged as versatile components in photocatalytic systems. CDs exhibit a unique combination of properties, including tunable optical absorption, rich surface chemistry, high photostability, and low toxicity.^22^ Importantly, CDs can be synthesized from abundant and renewable carbon sources using low energy and environmentally benign processes, aligning well with circular economy principles.^24–26^ When integrated with TiO_2_, CDs introduce multifunctional effects such as broadening of the light-harvesting capability of TiO_2_ and play a crucial role in regulating enhanced photogenerated charge-carrier dynamics.^27–35^ Owing to their favorable electronic structure and conductive nature, CDs act as efficient electron acceptors, or reservoirs, capturing photogenerated electrons from TiO_2_ and suppressing recombination.^30–32^ This behavior is largely enabled by the formation of strong interfacial interactions between CDs and TiO_2_, often involving Ti–O–C bonding, which facilitates rapid interfacial electron transfer and stabilizes the charge carriers.^35^ Consequently, CDs/TiO_2_ hybrid systems have been widely explored in various photocatalytic applications such as dye degradation, antibiotic remediation, hydrogen peroxide synthesis, carbon dioxide reduction, and hydrogen production.^26^ Despite this progress, a comprehensive understanding of how CDs regulate charge carrier dynamics at different CDs/TiO_2_ interfaces and influence catalytic rate enhancement remains an open challenge.

Although brookite is the less explored polymorph of TiO_2_ for photocatalytic hydrogen production, its unique orthorhombic crystal structure and distinct electronic properties make it attractive; therefore, brookite nanorods were selected for this study.^36,37^ To unravel the role of interfacial interaction, we designed two different hybrid systems: (i) CDs coupled with pristine brookite TiO_2_ nanorods (CDs/P–BTi) and (ii) CDs coupled with sonoreduced brookite TiO_2_ nanorods (CDs/R–BTi). Both systems were synthesized via a simple and green hydrothermal procedure with an optimized CD loading of 0.5 wt %. Comprehensive structural and surface analyses using HRTEM, X-ray photoelectron spectroscopy (XPS), X-ray diffraction (XRD), Raman spectroscopy, and BET measurements were carried out to understand how CDs form different interfacial heterojunctions with pristine and sonoreduced brookite TiO_2_ surfaces. Remarkably, CDs/R–BTi exhibits an excellent H_2_ production rate ∼669 μmol g^–1^ h^–1^ under 1 Sun illumination, which is 10-fold higher as compared to R–BTi. But CDs/P–BTi exhibits only 4-fold higher H_2_ production rate as compared to P–BTi. Such superior rate enhancement was achieved by coupling R–BTi with CD, which is mainly due to the enhanced photogenerated charge carrier separation. In-depth excitation–emission photoluminescence (PL) mapping combined with a mechanistic study confirms that CDs form strong Ti–O–C covalent bonds with oxygen vacancy-rich Ti^3+^ defect sites present on the surface of R–BTi through the oxygenated functional groups present on the CD surface. This robust heterojunction boosts photogenerated charge separation and their efficient migration to the respective catalytic centers in CDs/R–BTi, resulting in the abovementioned rate enhancements. In contrast, P–BTi does not form such strong bonding with CDs due to the absence of surface Ti^3+^ defect sites, causing inefficient charge separation. Collectively, these results demonstrate that utilizing an oxygen vacancy-rich TiO_2_ surface is a highly effective strategy for achieving multifold enhancement in photocatalytic H_2_ production rate as compared to the pristine TiO_2_ surface when CDs are used as cocatalysts, primarily by modulating interfacial bonding and charge-transfer dynamics.

## 2 Experimental Section

### 2.1 Synthesis of CDs/TiO_2_ Hybrid Systems

P–BTi and R–BTi were synthesized using our previously reported protocol.^38^ An aqueous stock solution of CDs was prepared following our earlier method.^39^

The CDs/P–BTi (or CDs/R–BTi) hybrid composites were synthesized by using a newly developed, simple hydrothermal process. First, DI water (5 mL) and ethanol (1.5 mL) were mixed, and then 100 mg P–BTi (or R–BTi) and 250 μL CD solution (2 mg mL^–1^, dispersed in water) were added in a sequence. To get a homogeneous suspension, the mixture was stirred for 5 h at ambient temperature. Afterward, the suspension was transferred into a 25 mL Teflon-lined stainless-steel autoclave and heated at 140 °C for 4 h. After the mixture was cooled to room temperature, the resulting CDs/P–BTi (or CDs/R–BTi) product was obtained by centrifugation, washing, and freeze-drying. Only by varying the amount of added CDs in the reaction mixture while keeping all other parameters fixed, composites with different CD loadings (0.25, 0.5, 1, 1.5, and 2 wt %) were prepared with P–BTi. Unless otherwise specified, both CDs/P–BTi and CDs/R–BTi refer to hybrid systems containing 0.5 wt % of CDs.

### 2.2 Photocatalytic Hydrogen Production

Photocatalytic experiments were carried out in a 21 mL quartz reactor. Two milligrams of the photocatalyst was added to a DI water: methanol solution (volume ratio 1:1) mixture. After 2 min of sonication, the solution was bubbled with Ar for 30 min to remove the dissolved oxygen. Then, the reactor was irradiated using a solar simulator equipped with a 150 W Xe arc lamp and an AM 1.5G filter (100 mW cm^–2^). The evolved hydrogen gas was collected with a gastight syringe and measured with a gas chromatograph GCMS-QP2010 SE (Shimadzu, Japan) and a TCD (thermal conductivity detector), using Ar as the carrier gas.

### 2.3 Characterization

XRD was performed at room temperature with an Empyrean diffractometer (PANalytical, Almelo, The Netherlands) in Bragg–Brentano geometry and using Co–Kα radiation (40 kV, 30 mA, λ = 0.1789 nm). The nanoscale-structural configuration of the synthesized samples was studied by transmission electron microscopy (TEM-JEOL 2010) with a LaB6-type emission gun, operating at 160 kV. Energy-dispersive X-ray spectroscopy mapping (energy-dispersive spectroscopy (EDS) mapping) and scanning transmission electron microscopy high-angle annular dark field (STEM HAADF) analysis were conducted using a HRTEM Titan G2 (FEI) instrument with an image corrector, operating at an accelerating voltage of 300 kV. Raman spectra were obtained using a DXR Raman spectrometer (Thermo Scientific, Massachusetts, USA). The ultraviolet–visible diffuse reflectance spectra (UV–vis DRS) were measured by using a Specord 250 plus (Analytik Jena, Jena, Germany) spectrophotometer. XPS measurements were performed on a PHI 5000 VersaProbe II (Physical Electronics, Chanhassen, USA) spectrometer using an Al Kα source (15 kV, 50 W). The obtained data were evaluated with the MultiPak software package (Ulvac-PHI Inc., Chigasaki, Japan). The binding energy value of the carbon C 1s line at 284.8 eV was taken as a reference for surface-charging corrections. BET (Brunauer–Emmett–Teller) measurements were carried out in a Quantachrome Autosorb iQ Station 3. Photoluminescence (PL) measurements were performed using an FLS980 fluorescence spectrometer (Edinburgh Instruments, Livingston, United Kingdom), equipped with an R928P photomultiplier tube (Hamamatsu, Japan). A 450 W xenon arc lamp was used as the excitation source for steady-state PL spectra.

## 3 Results and Discussion

### 3.1 Structural Characterization

The schematic diagram shown in Figure 1 represents the synthesis of two different types of photocatalysts: (a) CDs coupled with pristine brookite TiO_2_ nanorods (CDs/P–BTi) and (b) CDs coupled with sonoreduced brookite TiO_2_ nanorods (CDs/R–BTi). To achieve this, first, brookite TiO_2_ nanorods (P–BTi) were synthesized using a simple hydrothermal method. After the washing and drying steps, the nanorods were directly used for further modification. A probe sonication method was employed to produce sonoreduced brookite TiO_2_ nanorods (R–BTi). Under intense probe sonication, in aqueous solution, reactive species like H^•^, OH^•^, and hydroperoxyl (HO_2_^•^) radicals are generated. These species initiate a series of complex reactions leading to the formation of products such as H_2_O_2_, O_2_, or H_2_ which interact with the TiO_2_ nanocrystal surface, resulting in its surface modification and reduction.^40^ During this process, the color of P–BTi changed from white to black, confirming the successful formation of sonoreduced R–BTi, as shown in the inset photographs of Figure 1.

**Figure 1:**
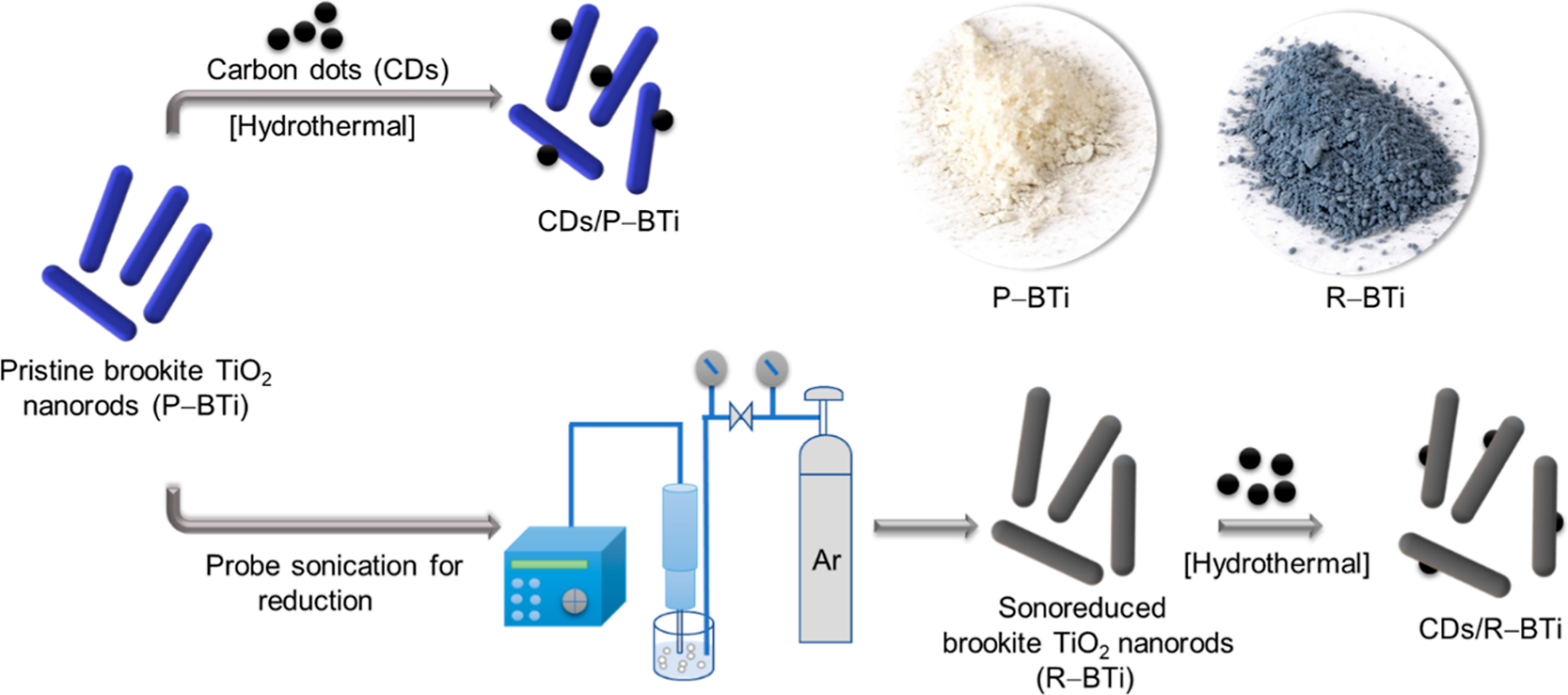
Schematic diagram depicting the synthesis of two hybrid systems used to compare photocatalytic hydrogen production rates: (a) CDs coupled with pristine brookite TiO_2_ nanorods (CDs/P–BTi) and (b) CDs coupled with sonoreduced brookite TiO_2_ nanorods (CDs/R–BTi). CDs are represented by black spheres, while CDs strongly bound within the surface cavities are depicted as black hemispheres. Inset photographs represent the color of P–BTi and R–BTi.

As shown in Figure S1a, the TEM image of the P–BTi sample reveals a uniform nanorod morphology with widths of 10–20 nm and lengths around 30–100 nm. Figure S1b presents an HRTEM image of a single nanorod, displaying well-defined lattice fringes with a *d*-spacing of 0.35 nm, corresponding to the (210) lattice plane of the brookite structure. The fast Fourier transform pattern of the selected area is also shown in the inset of Figure S1b, which confirms its highly crystalline nature. The morphology and size distribution of the sonoreduced R–BTi sample remain unchanged as compared to P–BTi (Figure S1c); however, a porous defect-rich surface is clearly observed (Figure S1d). In contrast to the conventional high-temperature reduction methods, where nanocrystals often undergo severe sintering process and turn into agglomerated bigger microcrystals, the sonoreduction preserves the original crystal facets and desired nanorod morphology, as confirmed by the HRTEM and SEM analyses in Figure S1.

To integrate CDs onto the surface of P–BTi and R–BTi (Figure 1), an identical hydrothermal procedure was carried out at 140 °C for 4 h in the presence of the same amount of CDs. Figure S2a presents a large-area TEM image confirming the spherical morphology of the as-synthesized CDs used in this study. The particle size histogram (Figure S2b) of CDs shows an average diameter of 3.4 nm, which is in good agreement with the previous report.^39^ After the washing and cleaning steps were performed, dried CDs/P–BTi and CDs/R–BTi powders were directly used for further characterization and photocatalytic reactions. The presence of CDs on the surface of P–BTi and R–BTi is confirmed by the HRTEM images of these two samples, as shown in Figure 2a,c respectively, which is crucial for this study. The interplanar lattice spacing of 0.21 nm was observed in the HRTEM images (Figure 2b,d) that correspond to the well-defined lattice spacings of the (100) plane of the graphite carbon core of the CDs.^26,29^ Notably, the highlighted red dashed line in Figure 2b clearly shows the presence of an ultrathin amorphous layer around the crystalline graphitic core of the CD. This amorphous shell mainly contains the oxygenated functional groups such as C–OH, −CHO, and −COOH.^22^ In the case of CDs/R–BTi, Figure 2d confirms that the CDs are embedded within the lattice of R–BTi forming a strong interfacial heterojunction. This unique coordination in CDs/R–BTi has been schematically highlighted in Figure 1.

**Figure 2:**
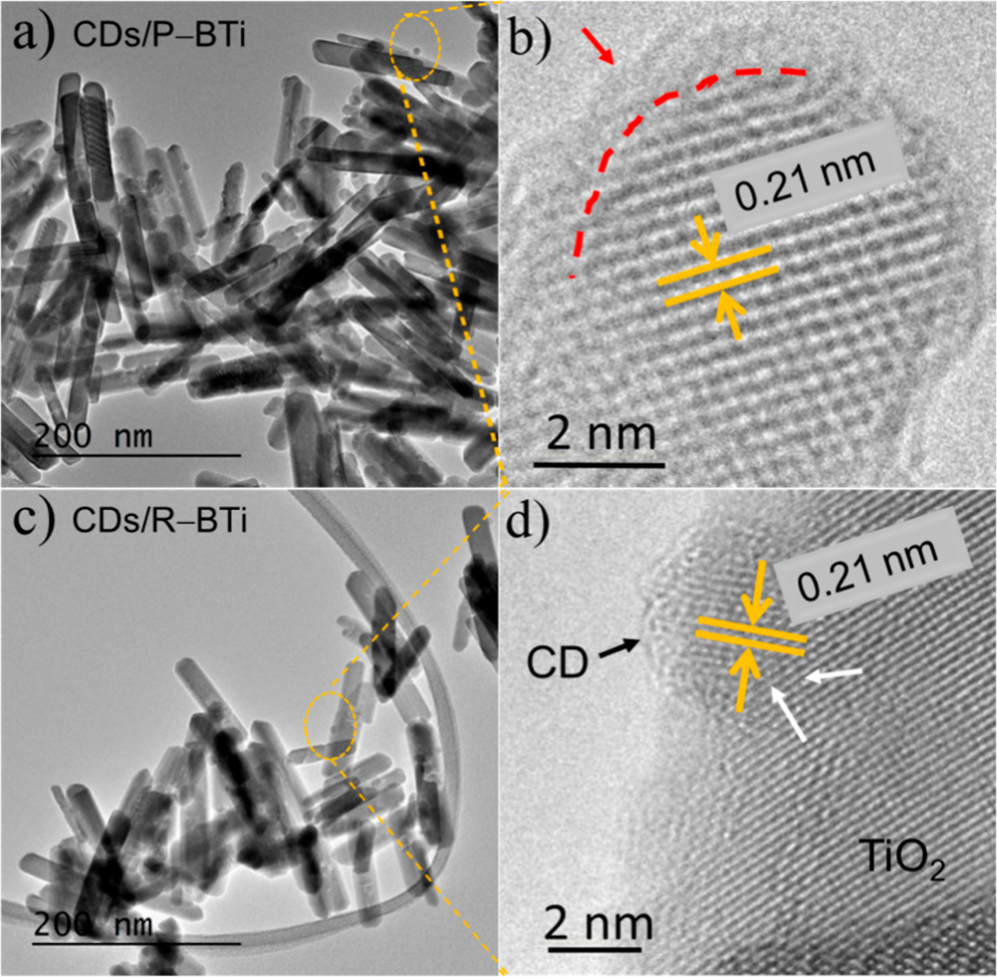
High-resolution TEM micrographs of the hybrid systems. (a) Large-area view of CDs/P–BTi and (b) magnified image of an individual surface-attached CD, showing a crystalline core with an amorphous shell (red arrow). (c) Large-area HRTEM image of CDs/R–BTi and (d) magnified HRTEM image of a CD confined in the lattice of R–BTi and exhibiting a hemispherical morphology. The white arrow indicates the formation of a strong interfacial heterojunction between the CDs and R–BTi.

High-angle annular dark-field scanning transmission electron microscopy (HAADF-STEM) was further employed to investigate the spatial distribution and dispersion of CDs in CDs/P–BTi and CDs/R–BTi (Figure 3). The HAADF-STEM images confirm the uniform nanorod morphology of TiO_2_ present in both samples. EDS elemental mapping of CDs/P–BTi (Figure 3a2–a4) reveals a homogeneous dispersion and intimate association of CDs on the surface of P–BTi, as highlighted by white arrows in Figure 3a4. The HAADF-STEM image of CDs/R–BTi (Figure 3b1) clearly shows the presence of surface defect-rich cavities on the R–BTi surface, generated during the sonoreduction procedure, which are also visible in Figure S1c. The corresponding EDS elemental mappings (Figure 3b2–b4) confirm that CDs are trapped within these surface cavities, as indicated by white arrows, also homogeneously distributed throughout the sample. The trapping of CDs at the defect-rich sites arises from strong interactions between the Ti^3+^ centers and oxygen-containing functional groups of the CDs, which will be discussed in the next section.

**Figure 3:**
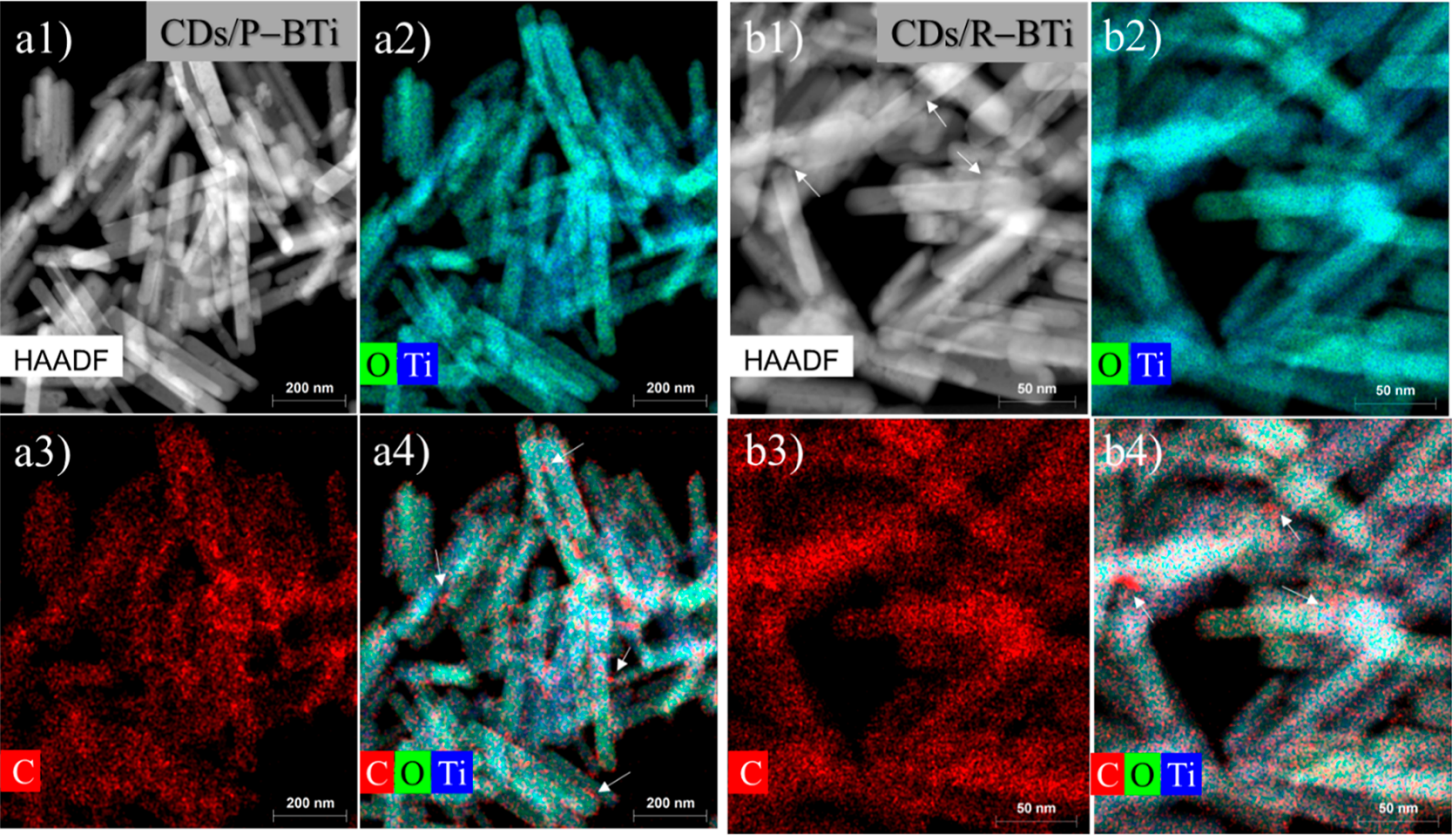
HAADF-STEM images and the corresponding EDS elemental maps of the hybrid systems. (a1) HAADF-STEM image of CDs/P–BTi and (a2–a4) corresponding EDS elemental mapping of carbon, oxygen, and titanium. CDs are homogeneously distributed on the P–BTi surface, as marked by white arrows in (a4). (b1) HAADF-STEM image of CDs/R–BTi, where white arrows highlight the presence of oxygen vacancy rich-cavities on the surface of R–BTi. (b2–b4) Corresponding EDS elemental mapping shows that the CDs are embedded within these surface cavities of R–BTi, as marked by white arrows in (b4).

### 3.2 Surface Analysis

In-depth XPS characterization was carried out for both samples, i.e., P–BTi and R–BTi, to understand the surface chemical composition. The survey spectra (Figure S3) confirm that titanium and oxygen are the dominant elements mainly present on the surface. High-resolution XPS measurements were performed in the Ti 2p region to characterize the Ti^4+^ and oxygen vacancy-related Ti^3+^ defect sites (Figure 4a,b). In Figure 4a, the Ti 2p HRXPS spectra confirm that P–BTi mainly contains Ti^4+^ oxidation state which exhibits two prominent peaks centered at 458.5 and 464.2 eV, corresponding to Ti^4+^2p_3/2_ and Ti^4+^ 2p_1/2_, respectively. The observed spin–orbit splitting difference for the Ti 2p doublet peaks in this system is 5.7 eV, which perfectly matches with the reported values for TiO_2_.^38^ Notably, as-synthesized P–BTi shows no detectable Ti^3+^ defect sites on its surface. After sonoreduction, the Ti 2p HRXPS spectra of R–BTi show that the main prominent peaks are centered at the Ti^4+^ region; however, two subpeaks appear in the Ti^3+^ region at 457.2 and 462.9 eV, corresponding to Ti^3+^2p_3/2_ and Ti^3+^2p_1/2_, respectively (Figure 4b). The emergence of these features indicates a higher fraction of oxygen vacancy-rich Ti^3+^ defect sites present on the surface of R–BTi.^41^ These defect sites are predominantly located within the surface cavities or pores, as also evidenced by the HAADF-STEM image in Figure 3b1. The EDS elemental mapping of CDs/R–BTi shows a strong C peak comparable with the O and Ti peaks, which further confirms that CDs are strongly coupled on the surface of CDs/R–BTi (Figure S4). To compare the chemical nature of carbon present in CDs/P–BTi and CDs/R–BTi, the HRXPS spectra of the C 1s region are shown in Figure 4c. The highlighted region starting from 286.4 to 290.5 eV (orange color) confirms the presence of surface-attached CDs in both samples. The dominant peak is centered at 284.7 eV, which is assigned to the C–C bonds in the graphitic carbon core of the CDs. The broad shoulder extended up to 290.5 eV confirms the presence of the oxygenated functional groups of CDs and indicates the formation of strong covalent bond through the Ti–O–C bond at the CDs/TiO_2_ interface.^35^ The Ti–O–C peak typically appearing at ∼286.0 eV is highlighted by a red dash line and is less pronounced due to low CD loading (0.5 wt %).^42^

**Figure 4:**
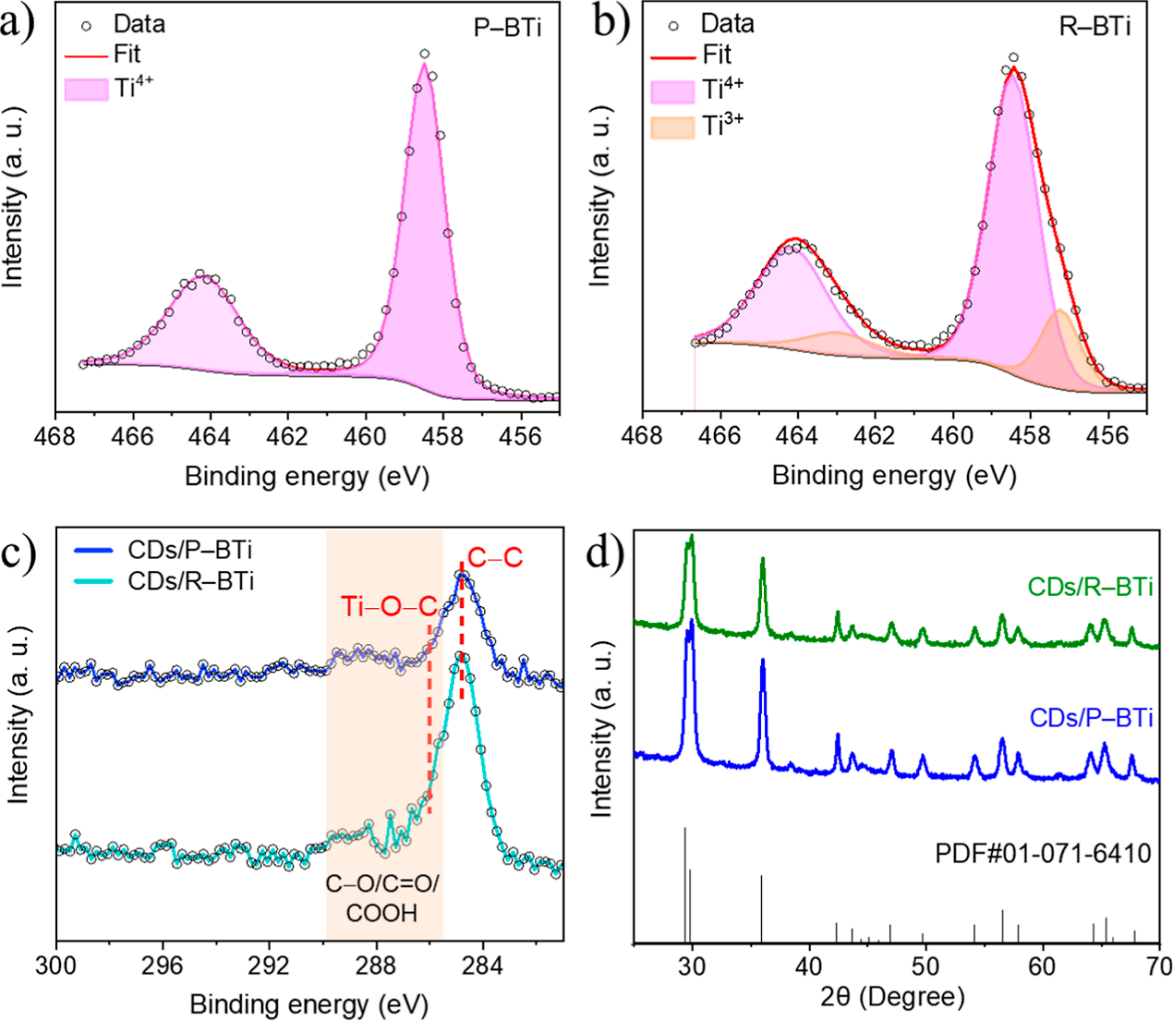
High-resolution XPS spectra of the Ti 2p region for (a) P–BTi and (b) R–BTi. (c) HRXPS spectra of the C 1s region to compare the nature of carbon present in CDs/P–BTi and CDs/R–BTi. (d) XRD patterns of CDs/P–BTi and CDs/R–BTi samples along with the reference pattern of brookite TiO_2_.

The hydrothermal decoration process is therefore crucial for promoting the formation of these robust Ti–O–C linkages, which play a key role in modifying the electronic structure and enhancing photogenerated charge separation.^26^ The crystallinity and phase composition of the hybrid systems were investigated using the powder XRD method, as shown in Figure 4d. All diffraction peaks can be indexed to the pure brookite phase of TiO_2_ (PDF no. 01-071-6410), with no detectable contribution from CDs due to their low loading.

The changes in the surface properties of the CDs/P–BTi and CDs/R–BTi hybrid systems were investigated by using the Brunauer–Emmett–Teller (BET) surface area analysis. The N_2_ adsorption–desorption isotherms of these samples are shown in Figure 5a,b, which typically exhibit type IV isotherms. The adsorption (blue) and desorption (red) curves do not overlap at a medium-to-high relative pressure, suggesting a clear hysteresis loop characteristic. The systematic variation in the BET specific surface areas is summarized in Figure 5c, providing clear insights into the effect of CD coordination on the surface of P–BTi and R–BTi. The as-synthesized P–BTi exhibits an initial specific surface area of 65 m^2^/g, and after sonoreduction, the R–BTi surface area increases to 75 m^2^/g. Notably, R–BTi (Figure S5) displays a type IV isotherm with a sharp capillary condensation step at high relative pressures (*P*/*P*_0_ ≈ 0.7–0.9) and H1-type hysteresis loops. This behavior indicates that the sonoreduction procedure introduces nonuniform pore sizes and a mesoporous structure in R–BTi, consistent with the HAADF-STEM observation (Figures 3b1 and S1c) and HRXPS results (Figure 4b). Upon loading CDs onto the surface of P–BTi, the specific surface area increases to 85 m^2^/g, which is due to the formation of an effective heterojunction in CDs/P–BTi. In contrast, the BET surface area decreases markedly from 75 to 67 m^2^/g after the formation of CDs/R–BTi hybrid nanostructures, as shown in Figure 5c. This decrease arises because, during the hydrothermal process, CDs become trapped within the surface pores or cavities of R–BTi through the formation of strong heterojunctions, as discussed earlier. Consequently, partial blockage of these cavities reduces the accessible surface area in CDs/R–BTi, as depicted schematically in Figure 5c. This interpretation is further supported by HAADF-STEM mapping, where CDs can be observed within the surface cavities of the R–BTi complex (Figure 3b4).

**Figure 5:**
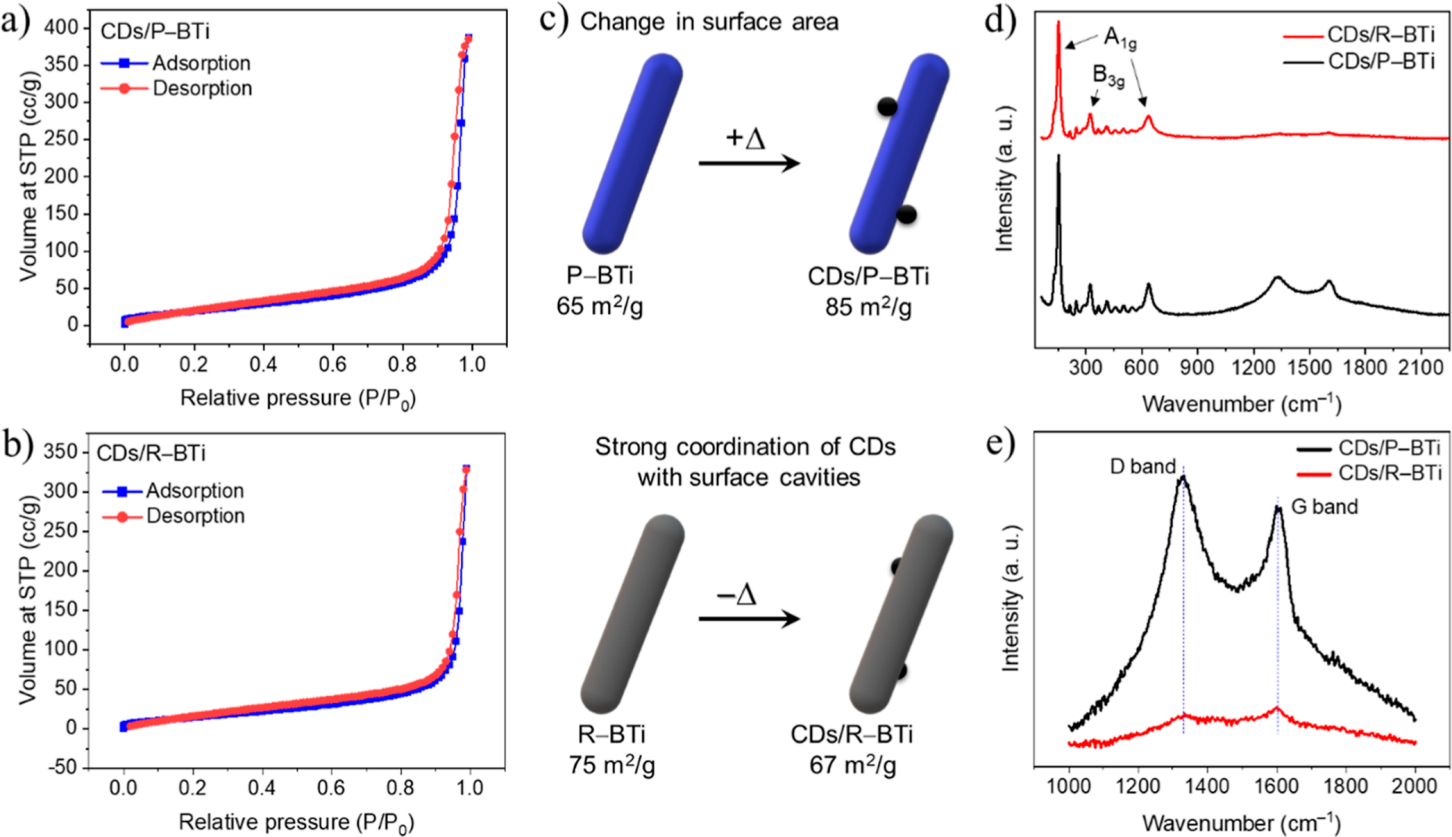
Nitrogen adsorption–desorption isotherms of (a) CDs/P–BTi and (b) CDs/R–BTi. (c) Schematic diagram illustrating the changes in surface area, upon coordination of CDs with different surfaces of TiO_2_. (d) Raman spectra of CDs/P–BTi and CDs/R–BTi. (e) Enlarged Raman spectra highlighting the characteristic signals of CDs in the hybrid nanostructures.

Raman spectroscopy is highly sensitive for the identification of different phases of TiO_2_ as well as the nature of carbon species present on the nanocrystal surface. Figure 5d shows the Raman spectra of the as-synthesized CDs/P–BTi and CDs/R–BTi hybrid systems. A strong Raman band at 155 cm^–1^ belongs to A1g vibrational mode, followed by the weaker bands in the range of 200–700 cm^–1^.^43^ Most prominent bands in this range can be assigned to B3g (322 cm^–1^) and A1g (631 cm^–1^) vibrational modes. This clearly confirms that both hybrid nanostructures contain the pure phase of brookite TiO_2_. Moreover, two additional peaks observed in the Raman spectra of both CDs/P–BTi and CDs/R–BTi samples at 1332 and 1605 cm^–1^ are ascribed to the D-band and G-band of carbon, respectively (Figure 5e).^25^ The D-band is associated with surface functionalization and disordered sp^3^-hybridized carbon, whereas the G-band originates from the vibration of sp^2^-bonded carbon atoms in a two-dimensional hexagonal lattice and corresponds to the E_2g_ mode of graphitic carbon. The relatively high ratio of *I*_D_/*I*_G_ ≈1.13 observed for CDs/P–BTi indicates the high density of surface defects in the CDs and suggests the formation of covalent bonds with the P–BTi surface during hydrothermal treatment. In contrast, the intensities of both the D- and G-bands in CDs/R–BTi significantly suppressed, while their peak positions remain unchanged. Abundant defect-rich mesopores in R–BTi provide more effective chemisorption sites for CDs, thus reducing their surface exposure along with interfacial charge redistribution due to strong hybridization, which significantly modify their electronic structures. Consequently, suppression and broadening of the D- and G-peak intensities are observed, as shown in Figure 5e. A comparison of the surface composition analysis by XPS confirms that both hybrid samples contain comparable amounts of CDs (Table S1), with no significant differences in loading or evidence of mass loss observed in the CDs/R–BTi sample.

### 3.3 Optical Properties and Photocatalytic Activity

To investigate the optical properties of these samples, UV–vis diffuse-reflectance spectroscopy (UV–Vis DRS) was carried out. As shown in Figure 6a, the sonoreduced R–BTi exhibits reduced reflectance (or higher absorption fraction) in the visible light region compared to P–BTi. This behavior can be attributed to an increased population of surface defects or oxygen vacancies in the lattice.^13^ The bluish gray color of R–BTi supports this observation, as shown in Figure 1. Both CDs/R–BTi and CDs/P–BTi samples show higher absorption fraction in the visible region as compared to their individual counterparts. This increase in light absorption property is due to the hybridization with CDs. UV–vis DRS combined with Tauc plot analysis is widely employed to determine the band gap energy (*E*_g_) of semiconductor nanomaterials (Figure 6b). The Tauc plot reveals no significant change in band gap energies among these four samples, with values ranging from ∼3.31 to 3.38 eV. These results indicate that all materials remain predominantly photoactive in the UV region of the solar light spectrum. CDs/R–BTi does not show any photocatalytic hydrogen production activity under visible light (λ > 420 nm) even though it strongly absorbs light in this region.

**Figure 6:**
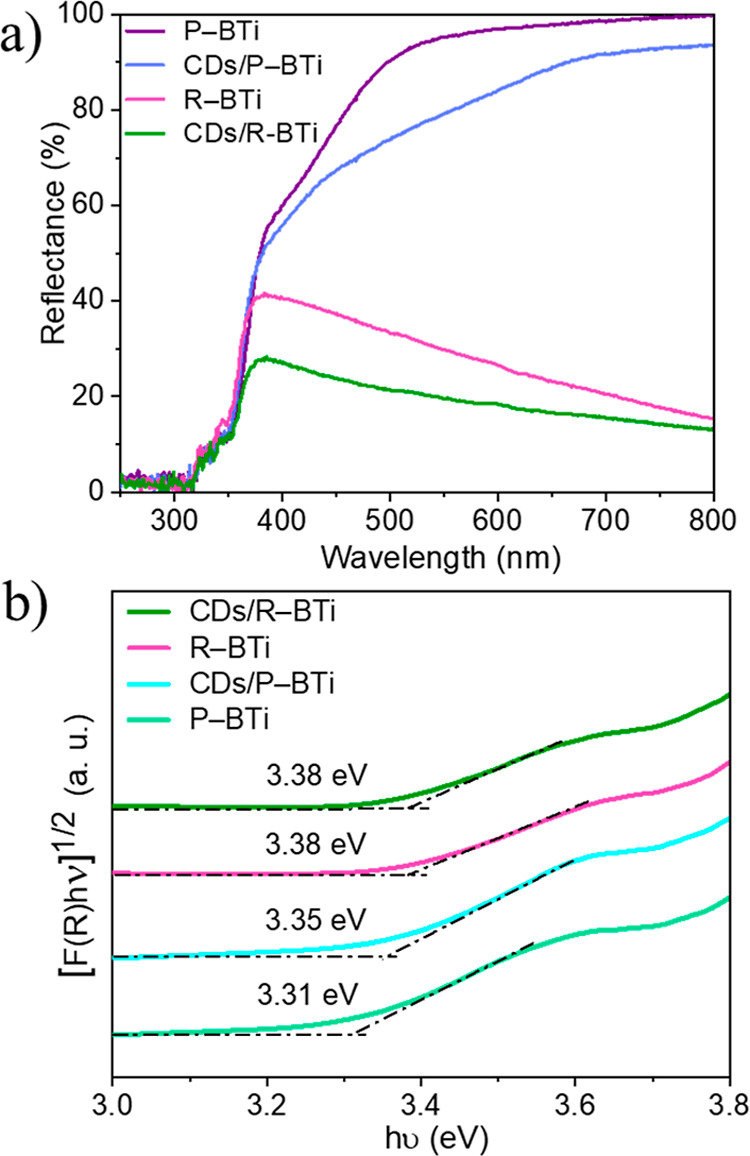
(a) UV–visible diffuse reflectance spectra of P–BTi, CDs/P–BTi, R–BTi, and CDs/R–BTi. (b) Calculation of band gap using the Tauc plot for these samples.

To understand the effect of different interfacial interactions between CDs and P–BTi or R–BTi on the photocatalytic H_2_ production rate, all experiments were carried out under standard conditions, i.e., under 100 mW cm^–2^ illumination in an aqueous methanol solution (1:1 v/v) at room temperature.^44^ As shown in Figure S6, P–BTi exhibits a relatively low photocatalytic H_2_ production rate of ∼19 μmol g^–1^ h^–1^. Pure CDs do not produce any H_2_ under identical experimental conditions, indicating that their energy levels are not suitable for driving this reaction independently. To investigate the coupling effect of CDs as a cocatalyst on P–BTi, a series of CDs/P–BTi photocatalysts were systematically synthesized with different loadings of CDs (Figure S6). A gradual increase in photocatalytic H_2_ production rate is observed as the CD content increases from 0.25 to 0.5 wt %. The highest H_2_ production rate of 74 μmol g^–1^ h^–1^ is achieved at a CD loading of 0.5 wt %, corresponding to an enhancement of approximately four times higher than that of P–BTi. However, further increases in CD loading led to a pronounced decrease in the H_2_ production rate. This decline is likely due to excessive CD coverage, which can induce light scattering and shielding effects, thereby reducing effective light absorption and the generation of photogenerated charge carriers. Accordingly, the optimized photocatalyst containing 0.5 wt % CDs was denoted as CDs/P–BTi. To investigate the effect of CD hybridization on sonoreduced R–BTi, CDs/R–BTi was prepared using the same synthetic protocol and identical CD loading. As shown in Figure 7a, the H_2_ production rate increases dramatically from 63 μmol g^–1^ h^–1^ (R–BTi) to 669 μmol g^–1^ h^–1^ for the CDs/R–BTi hybrid sample. Approximately 10 times, 35 times, and 9 times higher H_2_ production rates were achieved for CDs/R–BTi as compared to R–BTi, P–BTi and CDs/P–BTi, respectively. Such significant rate enhancement confirms enhanced photogenerated charge carrier separations due to the presence of Ti^3+^ defect sites and interfacial heterojunctions in CDs/R–BTi. Even after normalization of the H_2_ production rate with respect to the specific surface area, CDs/R–BTi remains the best-performing photocatalyst among the four samples, as shown in Figure 7b. Furthermore, Table S2 presents a comparative summary of the photocatalytic H_2_ production rates of CDs/TiO_2_ hybrid systems reported in the literature. It confirms that CDs/R–BTi outperforms other systems in terms of photocatalytic H_2_ production rate using only 0.5 wt % CDs as the cocatalyst and under 1 Sun illumination. Under 365 nm monochromatic UV-LED light irradiation, an AQY of 2.3% was obtained for CDs/R–BTi. The photocatalytic stability of CDs/R–BTi was evaluated through five consecutive cycling experiments (Figure 7c). The hybrid photocatalyst exhibits good long-term stability (Figure S7) with only a minor decrease in photocatalytic activity after each cycle. This slight decline can be attributed to unavoidable catalyst loss during Ar purging with a needle and/or the gradual accumulation of oxidized byproducts in the reaction solution over prolonged irradiation. From a practical perspective, the CDs/R–BTi hybrid consists of low-cost, earth-abundant, and nontoxic components, suggesting its potential for scalable production. However, a detailed techno-economic analysis is still required to evaluate its feasibility for future large-scale implementation.

**Figure 7:**
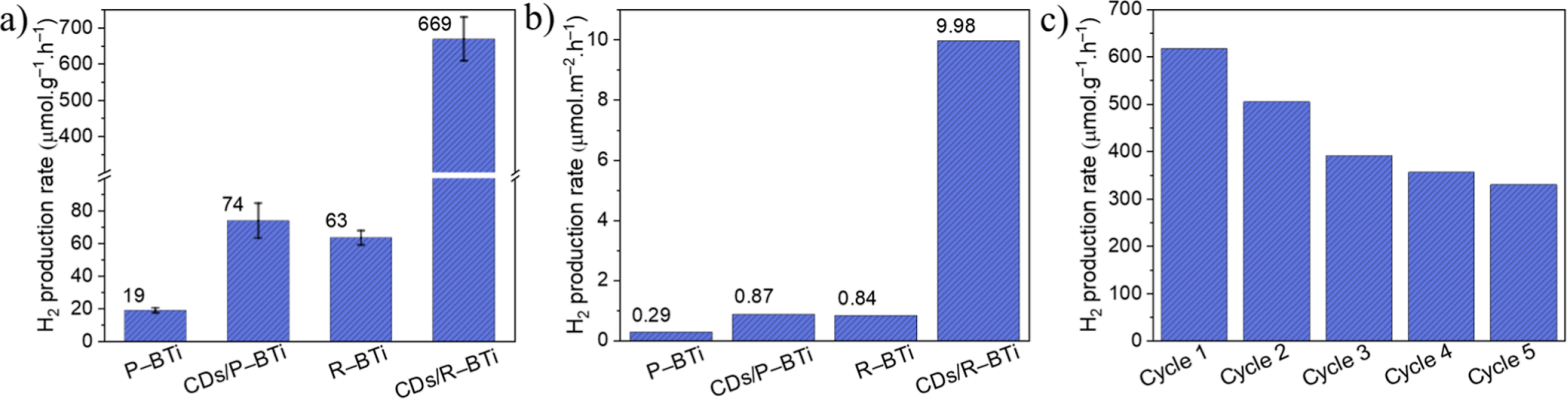
(a) Comparison of the H_2_ production rate of P–BTi, CDs/P–BTi, R–BTi, and CDs/R–BTi. The error bars are defined as standard deviation, and the center of each error bar represents the mean value of three independent experiments. (b) Surface area-normalized H_2_ production rates of the corresponding samples. (c) Long-term recyclability test of CDs/R–BTi.

### 3.4 Charge Carrier Dynamics and Mechanism

Excitation–emission photoluminescence (PL) maps were measured over a wide range of excitation wavelengths for P–BTi, R–BTi, CDs/P–BTi, and CDs/R–BTi (Figure 8a–d) to elucidate charge carrier dynamics. All PL maps were recorded at 80 K and under a N_2_ atmosphere to suppress thermally activated nonradiative processes. For P–BTi, a very strong PL emission band centered at 565 nm is observed, which is attributed to the oxygen vacancy-related defect states (Figure 8a).^45^ These defects originate from the high surface energy of exposed (210) facets in the nanorod morphology with a high density of low-coordinated surface atoms and a more open crystal structure. In contrast, R–BTi shows almost complete quenching of 565 nm emission (Figure 8b), indicating a substantial suppression of radiative electron–hole recombination. This behavior is attributed to the formation of abundant oxygen vacancies and Ti^3+^ defect sites induced by the sonoreduction process, which promotes efficient charge trapping and separation. Pure CDs show a characteristic PL emission in the deep violet region centered at 393 nm (Figure S8). Notably, this emission completely disappears in the CDs/P–BTi hybrid (Figure 8c), confirming the formation of an integrated hybrid system via the hydrothermal synthesis route. Simultaneously, the oxygen vacancy-related emission of P–BTi at 565 nm is significantly suppressed, indicating enhanced charge transfer and reduced recombination. Furthermore, CDs/P–BTi exhibits a broadened PL emission with a new maximum centered at approximately 480 nm, suggesting the emergence of new interfacial electronic states associated with CDs and P–BTi hybridization. In the case of CDs/R–BTi (Figure 8d), all PL emissions are completely quenched, evidencing highly efficient charge separation and nonradiative charge-transfer pathways enabled by the formation of strong interfacial heterojunctions with a modified electronic state. This superior suppression of charge recombination directly correlates with approximately 9-fold enhancement in H_2_ production rate, as observed for CDs/R–BTi compared to CDs/P–BTi.

**Figure 8:**
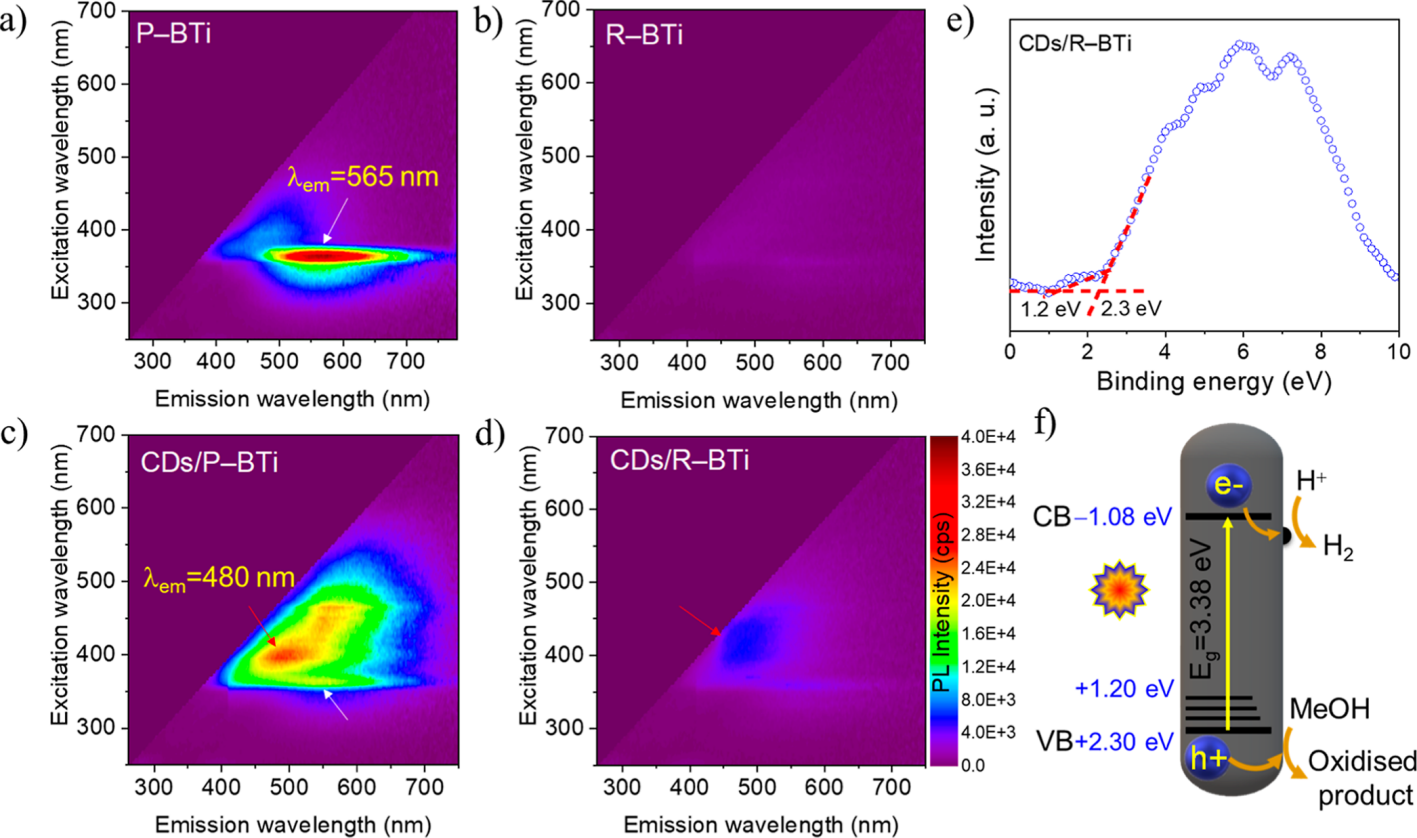
PL excitation–emission color maps of (a) P–BTi, (b) R–BTi, (c) CDs/P–BTi, and (d) CDs/R–BTi. (e) Valence-band XPS spectrum of CDs/R–BTi. (f) Schematic diagram for photocatalytic H_2_ production mechanism operating in CDs/R–BTi. R–BTi and CDs are depicted by gray nanorod and black hemispherical domain, respectively. VB, CB, and MeOH stand for the valence band, conduction band, and methanol, respectively.

Furthermore, the lifetimes of the charge carriers in both hybrid samples were investigated at a low temperature (*T* = 80 K) using time-resolved photoluminescence (TRPL) measurements monitored at an emission wavelength of 480 nm (Note S1). As shown in Figure S9, the PL decay curves were fitted with a three-exponential decay function. The average PL lifetimes (τ_ave_) for CDs/P–BTi and CDs/R–BTi are 2.9 and 4.2 ns, respectively. The higher τ_ave_ value observed for CDs/R–BTi compared to CDs/P–BTi indicates a reduced recombination rate of photogenerated electron–hole pairs, suggesting prolonged survival of the excited charge carriers and enhanced photocatalytic activity.

To obtain a more detailed band diagram in the high-performing CDs/R–BTi photocatalyst, valence band XPS (VB-XPS) was measured (Figure 8e). It shows a valence-band edge energy at ∼2.3 eV with a small tail extending up to 1.2 eV. Generally, it can be seen in the sample with abundant oxygen vacancies with Ti^3+^ defect sites, here, which is mainly produced by sonoreduction.^13^ Based on these results, a plausible band alignment and charge-transfer mechanism for CDs/R–BTi are proposed in Figure 8f. Upon photoexcitation, photogenerated electrons are efficiently transferred to strongly coordinated CDs from the CB of TiO_2_ via interfacial heterojunction. The CDs act as an electron accumulation center, facilitating the catalytic reduction of H^+^ to H_2_.^33^ On the other side, photoexcited holes get transferred to surface oxygen vacancy-rich Ti^3+^ defect sites and are subsequently quenched by the methanol molecules.^13,14,23,46^ The synergistic combination of efficient interfacial charge transfer, suppressed charge recombination, and spatial separation of redox sites endows the CDs/R–BTi system with markedly enhanced photocatalytic H_2_ production activity.^47–52^ These findings demonstrate that CDs function as an effective cocatalyst only when chemically bound to the defect-rich sonoreduced R–BTi system.

## 4 Conclusions

This work demonstrates, for the first time, the effect of different interfacial interactions of carbon dots (CDs) with pristine brookite TiO_2_ nanorods (P–BTi) vs sonoreduced brookite TiO_2_ nanorods (R–BTi) on the photocatalytic H_2_ production rate. A simple hydrothermal procedure was employed to synthesize two different types of hybrid photocatalysts: (i) CDs/P–BTi and (ii) CDs/R–BTi with 0.5 wt % CD loading. Comprehensive characterization and photocatalytic testing under 1 Sun intensity revealed that CDs/R–BTi shows an excellent H_2_ production rate ∼669 μmol g^–1^ h^–1^, which is 10 times, 35 times, and 9 times higher as compared to R–BTi, P–BTi, and CDs/P–BTi, respectively. Furthermore, CDs/R–BTi demonstrates a higher photocatalytic H_2_ production rate than the other comparable systems reported in the literature. The heterojunction-induced modification of the electronic state greatly promotes the separation and transport of photogenerated carriers to their respective catalytic centers in CDs/R–BTi. Raman spectra along with the excitation–emission photoluminescence (PL) maps further confirm this phenomenon. Overall, this work provides a new perspective on utilizing CDs as efficient, low-cost, and green cocatalysts for hydrogen production, particularly when integrated with reduced TiO_2_ systems. Such findings have potential applications in the further development of cheap, scalable, environmentally friendly solar-driven green hydrogen production technologies aligned with the sustainable energy objectives.

## Supplementary Material



## Notes

HRTEM results, XPS scan, BET isotherms, photoluminescence mapping, and comparison data for photocatalytic activity (PDF)
