# Supplementary material for: Oxygen Vacancy-Induced Strong Coordination of Carbon Dots with TiO2 for Enhanced Photocatalytic Hydrogen Production
Source: Energy Fuels. 2026 Apr 3;40(17):9630–40. doi: 10.1021/acs.energyfuels.6c00577 (PMC13137250; doi:10.1021/acs.energyfuels.6c00577)
Supplement: Supplementary file 1 [file ef6c00577_si_001.pdf]

Supplementary information

Oxygen Vacancy-Induced Strong Coordination of Carbon Dots with TiO_2 for Enhanced Photocatalytic Hydrogen Production

Mahdi Shahrezaei[1], Sergii Kalytchuk[1,2], Veronika Šedajová[1], Zdeněk Badura[1,2], Lukáš Zdražil[1,2], Morteza Afshar[1], Radek Zbořil[1,2], Štěpán Kment[1,2]* and Sourav Rej[1]*

[1] Regional Centre of Advanced Technologies and Materials, Czech Advanced Technology and Research Institute, Palacký University Olomouc, Šlechtitelů 241/27, 78371 Olomouc, Czech Republic.

[2] Nanotechnology Centre, Centre for Energy and Environmental Technologies, VSB–Technical University of Ostrava, 17. listopadu 2172/15, 708 00 Ostrava-Poruba, Czech Republic.

*Corresponding authors: stepan.kment@upol.cz; sourav.rej@upol.cz

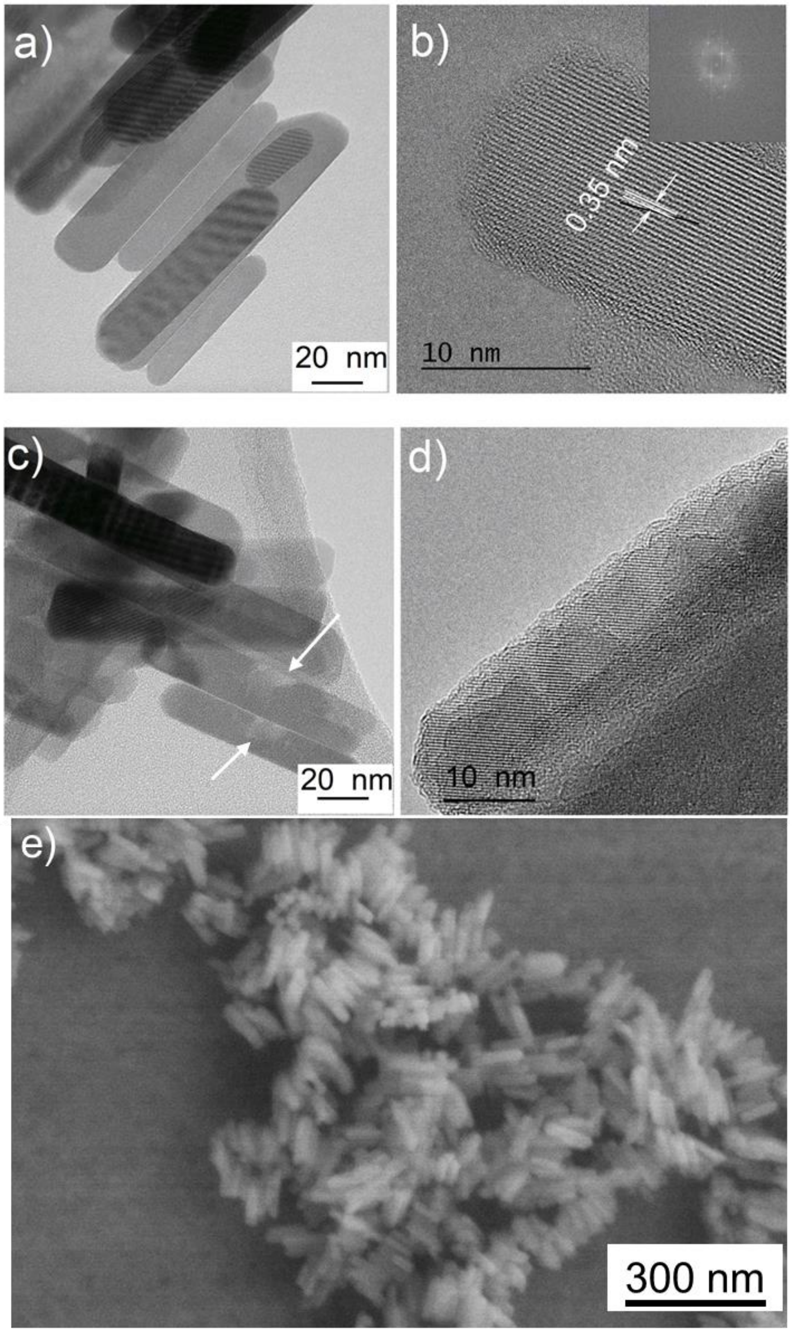

Figure S1. (a) TEM image of the P–BTi sample and (b) its corresponding HRTEM image. (c) TEM image of the R–BTi sample, where the white arrow indicates the porous sites on the surface and (d) its corresponding HRTEM image confirming its rough, defect-rich surface with pores. (e) Large area SEM image of R–BTi sample confirms high uniformity of size and shape.

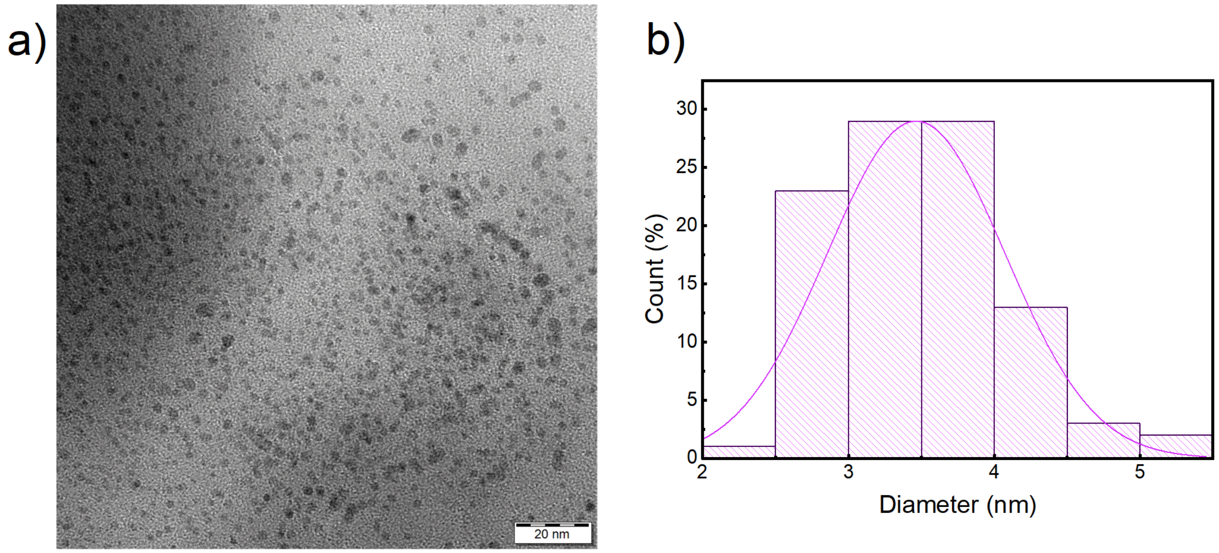

Figure S2. (a) Large area TEM image of CDs and (b) particle size distribution histogram as derived from TEM analysis for 100 particles.

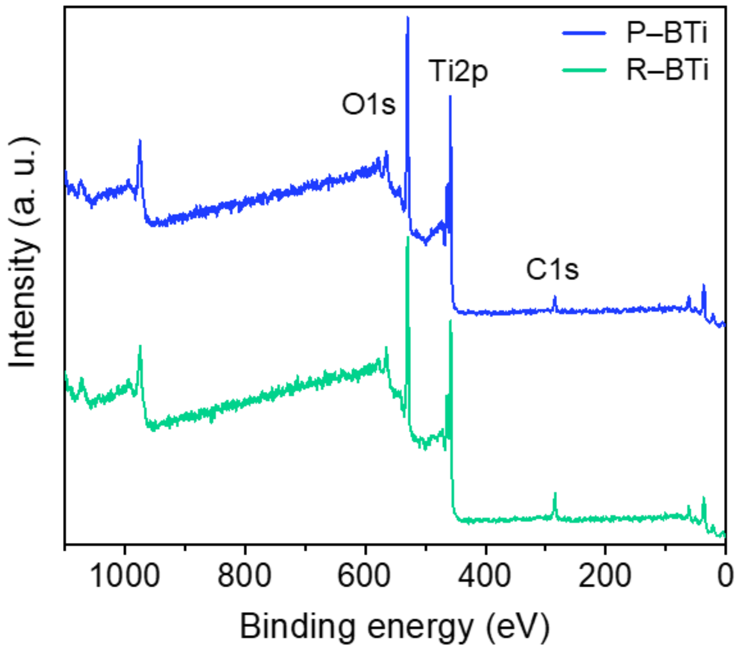

Figure S3. The XPS survey spectra of P−BTi and R−BTi samples.

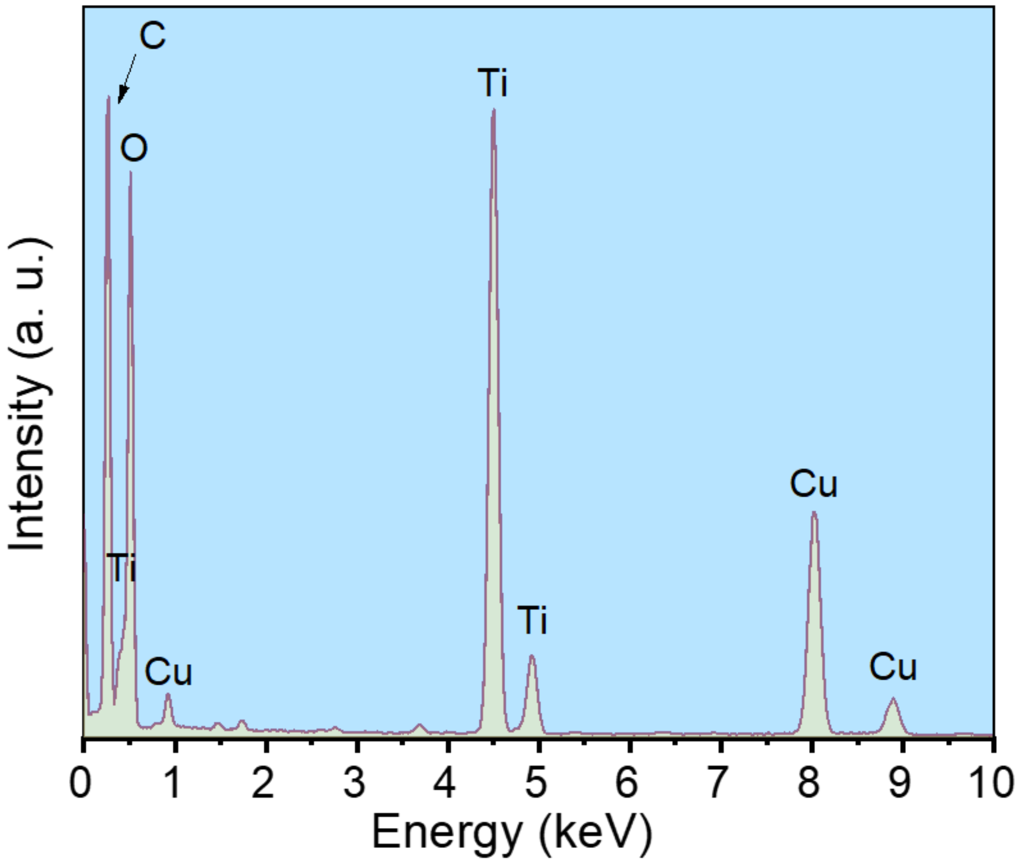

Figure S4. EDS spectrum of CDs/R–BTi. The pronounced carbon (C) signal confirms the presence of CDs on the surface of CDs/R–BTi. The signal of Cu comes from the TEM grid.

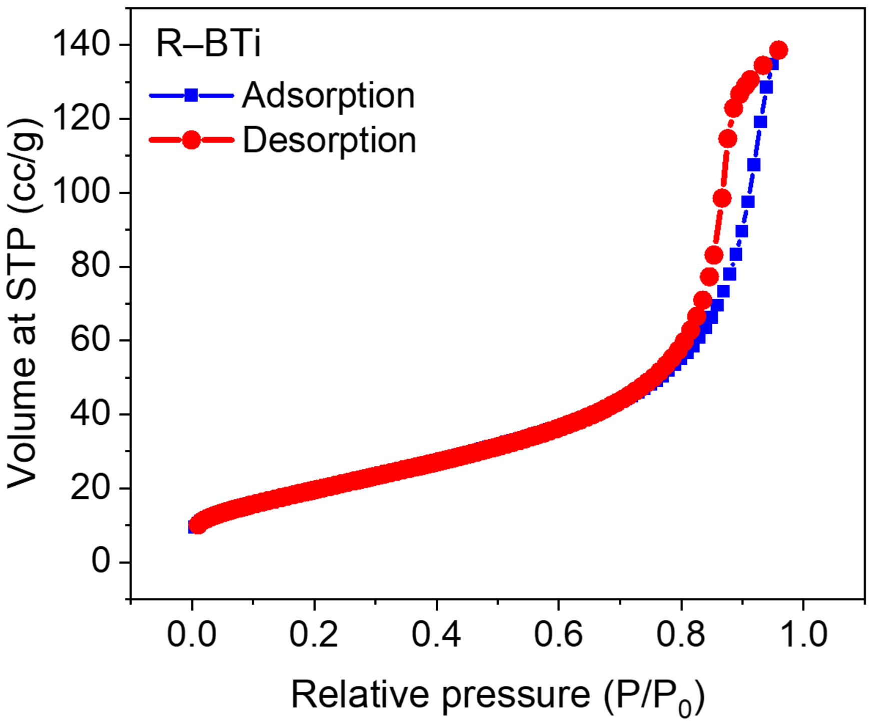

Figure S5. Nitrogen adsorption–desorption BET isotherm of R–BTi.

Table S1. Summary of surface concentrations of carbon, oxygen and titanium determined by XPS deconvolution.

**Photocatalyst**	**Carbon (at%)**	**Oxygen* (at%)**	**Titanium (at%)**
CDs/P−BTi	0.12	0.62	0.26
CDs/R−BTi	0.19	0.57	0.24

***** A drop in the at% of oxygen from CDs/P−BTi to CDs/R−BTi confirms creation of significant amount of oxygen vacancies in the R−BTi under sonoreduction condition.

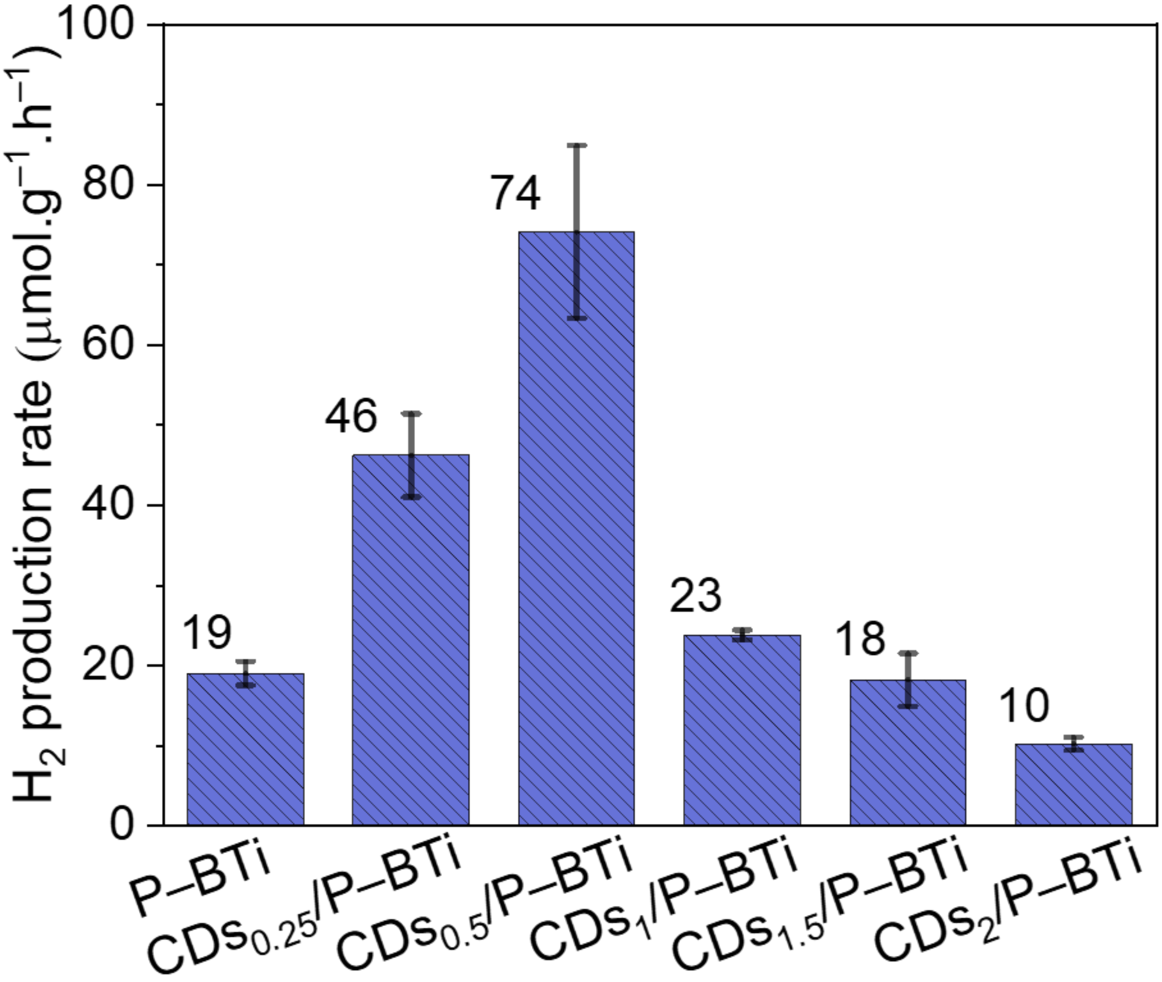

Figure S6. Photocatalytic H_2 production rate of the P–BTi decorated with varying loading of CDs.

Table S2. Summary of reported photocatalytic H_2 production performance using similar CDs/TiO_2 hybrid nanomaterials from the literature.

**Photocatalyst**	**Incident light (intensity)**	**Reactant solution**	**H_2 evolution rate (μmol g^{-1} h^{-1})**	**Reference**
CQDs/TiO_2	300 W Xe (not mentioned)	water/sodium sulfide+sodium sulfite	427 (AQY: not mentioned)	1
CQDs/TiO_2	AM 1.5 Solar simulator (Not mentioned)	water/Methanol	280 (AQY: not mentioned)	2
C-TiO_2	500 W Xe (135 mW cm^{-2})	water/sodium sulfide/sodium sulfite	162 (AQY: not mentioned)	3
CDs/TiO_2	300 W Xe (540 mW cm^{-2})	water/lactic acid	320.5 (AQY: not mentioned)	4
CQDs/TiO_2	PLS-SXE-300 (2.1 W cm^{-2})	water/methanol	2430 (AQY: not mentioned)	5
CQDs/TiO_2	500 W Halogen lamp (not mentioned)	water/methanol	182 (AQY: not mentioned)	6
Bio-CDs/TiO_2	300 W Xe (not mentioned)	water/methanol	603.92 (AQY: not mentioned)	7
1% Graphdiyne QDs-TiO_2	300 W Xe (not mentioned)	water/methanol	1322 (AQY: 3.87% at 400 nm)	8
CDs/R–BTi	150 W Xe arc lamp (100 mW cm^{-2})	water/methanol	669 (AQY: 2.3% at 365 nm)	**This work**

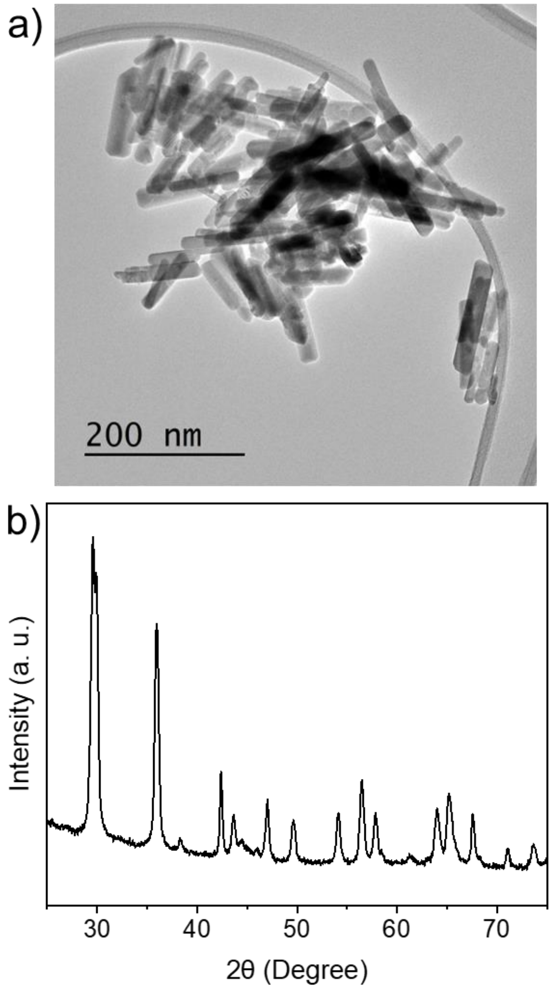

Figure S7. (a) TEM and (b) XRD of CDs/R–BTi after recyclability test, confirming its stability.

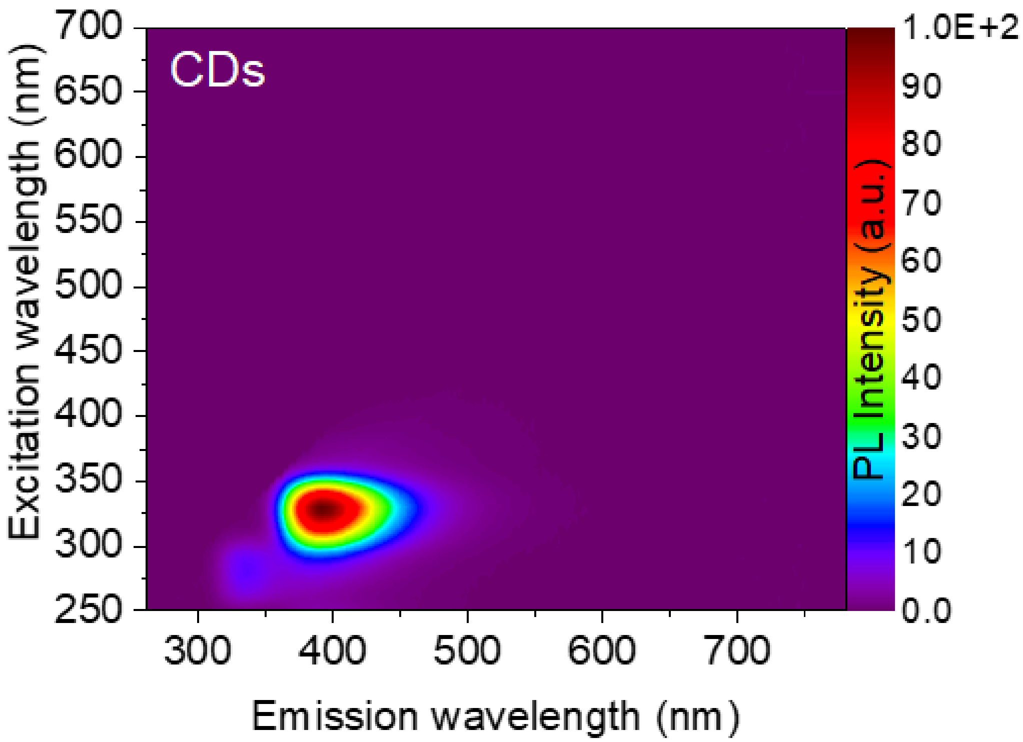

Figure S8. PL excitation-emission color maps of pure CDs.

Supporting Note 1: For time-resolved photoluminescence (TRPL) measurements, an EPL-375 pulsed diode laser (λ = 372 nm, pulse width 66.5 ps, repetition rate 20 MHz, average power 75 μW; Edinburgh Instruments) coupled with a time-correlated single photon counting (TCSPC) system was used. The obtained PL decay curves were fitted using a three-exponential function:

$$I(t) = \sum_{i=1}^{3} B_i \exp\left(-\frac{t}{\tau_i}\right), \ \sum_{i=1}^{3} B_i = 1,$$

where τ_i are the decay time constants, and B_i are the normalized amplitudes of the corresponding decay components. The intensity-weighted average decay lifetime, τ_{avg}, of the fluorescence decay was calculated according to:

$$\tau_{avg} = \frac{\sum_i B_i \tau_i^2}{\sum_i B_i \tau_i}$$

Powder samples were mounted on a front face sample holder using a dedicated quartz cell holder. Low temperature PL measurements were performed using a variable-temperature liquid nitrogen optical cryostat (OptistatDN2) controlled by a cryogenic programmable temperature controller (MercuryiTC, Oxford Instruments), providing a temperature stability of ±0.1 K (measured over 10 min). Time resolved PL spectra for different samples were recorded under identical instrumental conditions (identical excitation and emission band-passes and integration times).

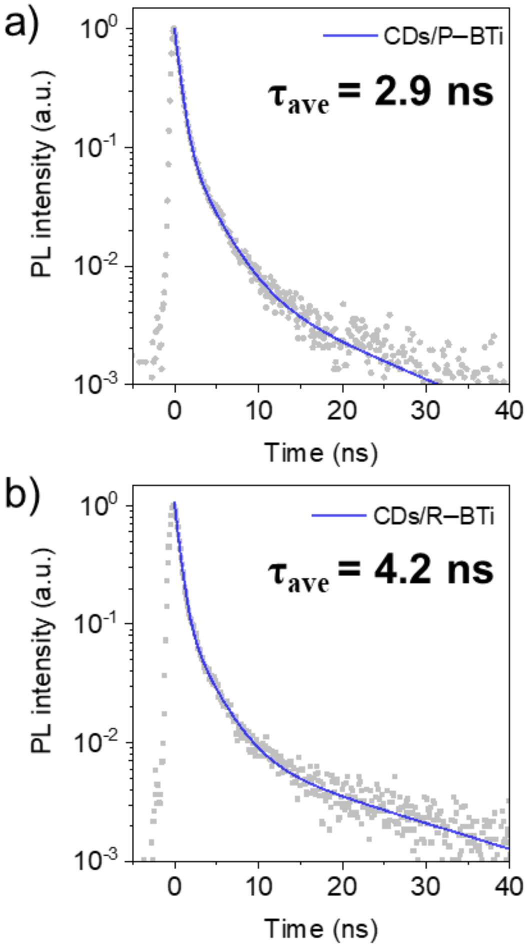

c)

Sample	B_1, %	τ_1, ns	B_2, %	τ_2, ns	B_3, %	τ_3, ns	τ_{ave}, ns
CDs/P−BTi	87.9	0.66	11.3	2.84	0.8	14.55	2.9
CDs/R−BTi	88.1	0.61	11.1	2.73	0.8	19.90	4.2

Figure S9. Time resolved PL spectra of (a) CDs/P−BTi and (b) CDs/R−BTi. (c) Fitting parameters of the TRPL decay spectra for CDs/P−BTi and CDs/R−BTi.

References:

(1) Yashwanth, H. J.; Rondiya, S. R.; Dzade, N. Y.; Hoye, R. L. Z.; Choudhary, R. J.; Phase, D. M.; Dhole, S. D.; Hareesh, K. Improved Photocatalytic Activity of TiO_2 Nanoparticles through Nitrogen and Phosphorus Co-Doped Carbon Quantum Dots: An Experimental and Theoretical Study. *Phys. Chem. Chem. Phys.* **2022**, *24* (25), 15271–15279.

(2) Tang, Y.; Hao, R.; Fu, Y.; Jiang, Y.; Zhang, X.; Pan, Q.; Jiang, B. Carbon Quantum Dot/Mixed Crystal TiO_2 Composites: Via a Hydrogenation Process: An Efficient Photocatalyst for the Hydrogen Evolution Reaction. *RSC Adv.* **2016**, *6* (99), 96803–96808.

(3) Zhang, X.; Sun, Y.; Cui, X.; Jiang, Z. Carbon-Incorporated TiO_2 Microspheres: Facile Flame Assisted Hydrolysis of Tetrabutyl Orthotitanate and Photocatalytic Hydrogen Production. *Int. J. Hydrogen Energy* **2012**, *37* (2), 1356–1365.

(4) Zhou, X.; Chen, D.; Li, T.; Chen, X.; Zhu, L. Pd and Carbon Quantum Dots Co-Decorated TiO_2 Nanosheets for Enhanced Photocatalytic H_2 Production and Reaction Mechanism. *Int. J. Hydrogen Energy* **2024**, *53*, 1361–1372.

(5) Zhao, H.; Yu, X.; Li, C. F.; Yu, W.; Wang, A.; Hu, Z. Y.; Larter, S.; Li, Y.; Golam Kibria, M.; Hu, J. Carbon Quantum Dots Modified TiO_2 Composites for Hydrogen Production and Selective Glucose Photoreforming. *J. Energy Chem.* **2022**, *64*, 201–208.

(6) Yu, H.; Zhao, Y.; Zhou, C.; Shang, L.; Peng, Y.; Cao, Y.; Wu, L. Z.; Tung, C. H.; Zhang, T. Carbon Quantum Dots/TiO_2 Composites for Efficient Photocatalytic Hydrogen Evolution. *J. Mater. Chem. A* **2014**, *2* (10), 3344–3351.

(7) Huang, X.; Sun, L.; Liu, X.; Ge, M.; Zhao, B.; Bai, Y.; Wang, Y.; Han, S.; Li, Y.; Han, Y.; Zhang, C. Increase and Enrichment of Active Electrons by Carbon Dots Induced to Improve TiO_2 Photocatalytic Hydrogen Production Activity. *Appl. Surf. Sci.* **2023**, *630*, 157494.

(8) Ullah, W.; Slassi, A.; Wang, C.; Paineau, E.; Ha-Thi, M. H.; Pino, T.; Halime, Z.; Gayral, A.; Vallet, M.; Degrouard, J.; Cornil, J.; Ghazzal, M. N. Defect-Rich Graphdiyne Quantum Dots as Efficient Electron-Donors for Hydrogen Generation. *Adv. Energy. Mater.* **2024**, *14* (30), 2401547.
